# Towards a comprehensive global approach to prevention and control of NCDs

**DOI:** 10.1186/s12992-014-0074-8

**Published:** 2014-10-28

**Authors:** Martin McKee, Andy Haines, Shah Ebrahim, Peter Lamptey, Mauricio L Barreto, Don Matheson, Helen L Walls, Sunia Foliaki, J Jaime Miranda, Oyun Chimeddamba, Luis Garcia-Marcos, Paolo Vineis, Neil Pearce

**Affiliations:** European Centre on Health of Societies in Transition (ECOHOST), London School of Hygiene and Tropical Medicine, London, WC1H 9SH UK; Departments of Social and Environmental Health Research and of Global Health and Development, London School of Hygiene and Tropical Medicine, London, UK; Centre for Global NCDs, London School of Hygiene and Tropical Medicine, London, UK; Instituto de Saude Coletiva, Federal University of Bahia, Bahia, Brazil; Centre for Public Health Research, Massey University, Wellington, New Zealand; Leverhulme Centre for Integrative Research on Agriculture and Health, London, UK; National Centre for Epidemiology and Population Health, The Australian National University, Canberra, Australia; CRONICAS Centre of Excellence in Chronic Diseases, and School of Medicine, Universidad Peruana Cayetano Heredia, Lima, Peru; Department of Epidemiology and Preventive Medicine, School of Public Health and Preventive Medicine (SPHPM), Monash University, Melbourne, Australia; Respiratory and Allergy Units, Arrixaca University Children’s Hospital, University of Murcia and IMIB-Arrixaca Research Institute, Murcia, Spain; MRC-PHE Center for Environment and Health, School of Public Health, Imperial College, London, UK

**Keywords:** Non-communicable diseases, Prevention, Health systems

## Abstract

**Background:**

The “25×25” strategy to tackle the global challenge of non-communicable diseases takes a traditional approach, concentrating on a few diseases and their immediate risk factors.

**Discussion:**

We propose elements of a comprehensive strategy to address NCDs that takes account of the evolving social, economic, environmental and health care contexts, while developing mechanisms to respond effectively to local patterns of disease. Principles that underpin the comprehensive strategy include: (a) a balance between measures that address health at the individual and population level; (b) the need to identify evidence-based feasible and effective approaches tailored to low and middle income countries rather than exporting questionable strategies developed in high income countries; (c) developing primary health care as a universal framework to support prevention and treatment; (d) ensuring the ability to respond in real time to the complex adaptive behaviours of the global food, tobacco, alcohol and transport industries; (e) integrating evidence-based, cost-effective, and affordable approaches within the post-2015 sustainable development agenda; (f) determination of a set of priorities based on the NCD burden within each country, taking account of what it can afford, including the level of available development assistance; and (g) change from a universal “one-size fits all” approach of relatively simple prevention oriented approaches to more comprehensive multi-sectoral and development-oriented approaches which address both health systems and the determinants of NCD risk factors.

**Summary:**

The 25×25 is approach is absolutely necessary but insufficient to tackle the the NCD disease burden of mortality and morbidity. A more comprehensive approach is recommended.

## Background

In 2011 world leaders met at the United Nations (UN) to state their commitment to “to address the prevention and control of non-communicable diseases worldwide” [1]. The World Health Organization, as the UN’s specialised agency for health, subsequently published its Global NCD Action Plan 2013–2020 [2,3]. This proposes a series of voluntary targets to tackle the emerging global epidemic of Non-Communicable Diseases (NCDs) [4,5], with the goal of achieving a 25% relative reduction in mortality from four conditions (cardiovascular disease, cancer, diabetes and chronic respiratory diseases) by 2025 [6]. The targets to achieve this “25×25” strategy [7] include reducing mortality from these four conditions and halting the rise in diabetes and obesity, by reducing alcohol consumption, increasing physical activity, reducing dietary salt and smoking, improving blood pressure control, and enhancing treatment of those at risk from or suffering from the major NCDs [8]. Yet, as we have argued elsewhere, these responses, which might be considered the ‘standard’ model, are modest given the scale of the challenge [9]. They will be insufficient to respond to the global forces driving the epidemics of NCDs, in particular the tobacco, alcohol and food industries, they are limited to four main NCDs which together account for only 54% of NCD DALYS, and they pay insufficient attention to the non-traditional risk factors, such as air pollution, and the need to strengthen health systems. In this paper we argue for the need to go beyond this standard model and discuss seven key issues that a more comprehensive approach should consider.

## Discussion

### Balancing collective and individual responses

A comprehensive strategy should include an appropriate balance of actions to prevent disease acting at the individual and the collective and the local and the global levels. All are important; there will always be a need for individual-level interventions, especially for those at high risk or with established disease, while there are some determinants of disease, such as air pollution and inadequate water supply that can only be tackled by collective action. However, interventions at the population level often achieve much greater benefits at lower cost. Similarly, policies must be tailored to local contexts while tackling shared regional or global threats.

The need for balance between the individual and the collective can be illustrated with tobacco-attributable disease [10]. At an individual level, existing smokers can be helped with individual or group behavioural interventions. Yet the main drivers of both smoking initiation and cessation lie at the population level, related to price, availability and marketing. Thus, increases in cigarette prices are effective in reducing smoking, [11] including in low- and middle-income countries [12]. Bans of point-of-sale displays reduce perceived availability [13] and bans on advertising in print media, radio and television serve to de-normalise the act of smoking [14]. This is further encouraged by removal of the one place where advertising is still permitted in many countries, the packs themselves, with emerging data from Australia on how standardised packaging reduces the attractiveness of smoking to young people [15]. The population-level interventions are either cost neutral or, in the case of tax rises, revenue raising, while individual approaches always incur costs associated with their delivery. The same principles apply to reducing hazardous consumption of alcohol and certain food products. Thus, density of alcohol [16] outlets is an important determinant of consumption while increases in alcohol prices reduce hazardous consumption [17]. It is, however, important to note that those selling these products will always argue against legislation and in favour of voluntary agreements [18], precisely because the latter are known to be less effective unless they incorporate clear targets and robust independent monitoring, which is rarely the case [19].

Collective measures often require action against “structural factors” or the “causes of the causes” [20] of disease. These will require measures in areas as diverse as tax and welfare, housing, transport, industry and agriculture, sometimes brought together under the heading of “Health in all Policies” [21]. This demands understanding of the complex and interrelated network of economic, environmental, social, commercial, and cultural determinants involved in health and the need for a range of integrated actions involving all parts of government and not just health ministries [22]. Although some actions to tackle these determinants can be taken at the local or national level, many will require concerted international responses.

Developing effective collective responses will be challenging as, in many countries, and especially where governance is weak and transparency limited, health ministries are the weakest within government and face opposition from powerful vested interests with close links to other stronger ministries and are often excluded from international discussions on key areas such as trade.

### What works where?

The elements of a comprehensive strategy should be appropriate to the settings in which they are applied. As noted in the WHO Action Plan [2], it cannot be assumed that technologies or policies developed in high-income settings, where much of the evidence comes from, can simply be transferred to low and middle-income countries (LMIC), where the burden of disease is rising rapidly. These approaches will often require unaffordable levels of funding, skilled workers who do not exist, managerial systems that are already struggling, and distribution systems unable to ensure regular supplies of essential medicines [23]. Moreover, transfer of policies must also take account of cultural norms. Thus, in many countries in Asia, attempts to control hypertension must consider traditional belief systems, such as those found in India, China, and South East Asia, whereby “dis-ease” is equated with pain and feeling ill and there is limited recognition of the importance of treating an asymptomatic risk factor [24]. It must also take account of affordability of both treatment and the means of obtaining it (such as transport costs), in countries where even a miniscule charge may be unaffordable to families already trapped in a cycle of debt [25].

Nor can it be assumed that policies developed in one LMIC setting can be transferred to another at a similar level of development, given the need to take account of differences in implementation capacity, shared understanding of goals among those making and those implementing policies and, sometimes corruption [26]. The experience of scaling up HIV/AIDS services highlights the problems that arise when applying costing data derived in one setting to another where local data are lacking [27].

There is an extensive literature on international lesson learning and policy transfer [28] and approaches such as “theory driven evaluation” [29] or “realist evaluation” [30] go beyond the question whether something works but rather to understand circumstances that make it work properly. Although these approaches are used increasingly in health policy research [31], many health-related initiatives still ignore the context in which they are to be applied.

For these reasons, it is essential to build a portfolio of evidence on the cultural appropriateness, cost effectiveness, and equity implications in different settings, based on the creation of a culture of evaluation that runs through the policy process, from generating locally relevant evidence to translating it into formats that can be used by policy makers, drawing especially on the emerging body of evidence on knowledge translation in low and middle income countries [32].

### Strong primary health care

As noted in the WHO Action Plan [2], action against NCDS should build on a foundation of strong primary care. In 1978, in Alma Ata, 134 countries committed to primary health care, defined as “the first level of contact of individuals, the family and community with the national health system bringing health care as close as possible to where people live and work, and constituting the first element of a continuing health care process” [33]. However, in over three decades since then, few health systems have lived up to that commitment. Instead, often encouraged by donors, many have invested in vertical health programmes, targeting individual conditions, with AIDS, tuberculosis, malaria, and more recently maternal health attracting particular attention. Yet research on health systems in countries at all levels of development shows that integrated primary health care provides more effective care and at lower cost than more fragmented systems [34] and countries that have adopted primary health care achieve better health outcomes [35-37].

The common risk factors for NCDs increase the probability of multiple disorders. Poor diet increases the risk of risk of diabetes, cardiovascular disease and some cancers, while smoking increases the risk of chronic obstructive pulmonary disease, cardiovascular disease, and many cancers. Moreover, risk factors often cluster within populations, disproportionately affecting those living in deprived circumstances [38]. There is also an increasing recognition of the growing rates of multi-morbidity in ageing populations, including not just the four diseases mentioned in the 25×25 strategy but also a wide range of conditions such as mental illness and neurological and musculo-skeletal diseases [39]. Crucially, many patients with chronic infectious disease, such as tuberculosis or HIV/AIDS, have co-existent NCDs [40,41]. There is little point in treating one condition but leaving the patient with the consequences of several others.

An effective response must involve the provision of integrated health services, as close to the population as possible, with no financial barriers to access. Yet this is often far from reality. For example, people with type I diabetes in many LMIC die because of lack of insulin [42]. In a 2003 study from Mozambique it was estimated that a child with type 1 diabetes could expect to live 3.8 years in Maputo, the capital, but only 7 months in rural areas [43]. Hypertension is also poorly controlled in many countries, with one recent study finding that only 12.7 of those with hypertension are aware of it, have received treatment, and achieved control, [44], leading to much avoidable death and disability. These challenges can only be addressed by a strong primary care system that can identify and treat those in need in a timely manner and, where necessary, refer them for more specialised care.

### Emerging threats

The 25×25 strategy addresses the traditional risk factors, but not the underlying drivers of them. Just as mosquitoes are the vectors of the micro-organisms causing some devastating communicable diseases, it is now recognised that major corporations spread the traditional risk factors for NCDS [45]. Trade liberalisation, itself a major goal of these corporations that can exert a powerful influence on international regulations, is associated with increases in a range of NCDs [46]. These corporations can move extremely quickly, as seen when once-closed economies, such as countries emerging from the USSR in the 1990s [47] and more recently, Myanmar, open to the world. They exploit any regulatory gaps, for example by placing brand imagery on consumer goods (brand stretching) or using social media to circumvent advertising bans. The major tobacco companies have exploited the opportunities presented by electronic cigarettes to reposition themselves as partners with governments in the fight against tobacco, while using the ability to advertise these new products to promote imagery that glamorises smoking [48]. Unfortunately, legislative processes are often very slow, and are slowed even further by corporate pressure, as with the European Union’s revision of the Tobacco Products Directive [49]. Consequently, there is a strong case for a global horizon scanning function, identifying tactics used to promote unhealthy products, coupled with support for rapid legislative responses, such as a repository for existing legislation that can be adapted by other countries [50]. The alternative is that corporations will write the legislation, as happened when the tobacco industry assisted former Soviet countries with tax codes that, unsurprisingly, were the most favourable for their products. Effective action cannot ignore the political roots of global health, including what Kickbusch has termed the “commercial determinants of health” [51], demanding effective global governance based on commitments to global solidarity and shared responsibility [52].

### The post-2015 agenda

The global response to NCDs should not stand in isolation from other international processes. The post-2015 development agenda represents an important opportunity to integrate efforts to reduce the burden of NCDs and promote sustainable development [3]. The recent identification of NCDs as a major threat to the global economy [53,54] provides a lever for moving NCDs from a peripheral to a central concern of global development. It now seems likely that Universal Health Coverage will feature prominently in the post-2015 development agenda [55]. On 12 December 2012 it was endorsed unequivocally by the UN General Assembly (including the United States), which resolved that it confirmed the “intrinsic role of health in achieving international sustainable development goals” [56]. Universal Health Coverage has been defined by the WHO as “access to key promotive, preventive, curative and rehabilitative health interventions for all at an affordable cost….”, subject to the proviso that the cost of care “…[should] not put people at risk of financial catastrophe” [57]. Making it a reality will require the implementation of evidence-based, cost-effective, and affordable approaches that can reduce the burden of NCDs and promote sustainable development [3,58].

The driving forces behind important risk factors for NCDs, such as fine particulate air pollution , physical inactivity and unhealthy diets, are linked to current patterns of unsustainable development, including the combustion of fossil fuels which results in climate change [58]. Indicators for the post 2015 development agenda must therefore link health and sustainability, addressing sectors such as energy, transport, housing, food and agriculture [59] as well as reflecting progress towards Universal Health Coverage. However, current proposals fall far short of what is needed [60].

### Matching priorities to resources

An effective strategy must take account of the challenges that each country faces and its access to resources. Each country is at a different stage in the epidemiological transition. Its burden of NCDs is influenced by the demographic composition of its population, its exposure to risk factors, its stage in the nutritional and epidemiological transitions, its geography and its ability to provide effective health care [61]. For example, smoking-related disease may be less in some countries because of cultural barriers to smoking and limited penetration by tobacco corporations. While the debate about the role of thrifty genotypes and phenotypes remains unresolved [62], it is clear that some populations are much more susceptible to diabetes than others. In many countries, diet continues to be influenced heavily by traditional patterns of agriculture, even if this is rapidly changing. Moreover, these differences are seen not only among countries but within them, with substantial regional differences in large countries such as Brazil, India, or China, and between rural and urban areas almost everywhere, with the NCD epidemic progressing most rapidly in the latter.

Each country also has differing levels of available resources, whether raised domestically or from development assistance. Consequently, it will be essential that strategies to tackle NCDs are matched to both the resources available and the burden of disease, while not forgetting the need to ensure equity. There is a critical need to strengthen capacity in health ministries, which often have a much lower status and priority than other ministries, such as trade and defence. This will require a comprehensive package of measures to recruit, develop and retain skilled analysts and policy experts, supported by access to information about both their own country and the international literature on effective interventions. This, in turn, will require new tools for surveillance of NCDs and their risk factors, so far, these have been largely missing from surveys such as the Demographic and Health Surveys that have concentrated on maternal and child health and, more recently, HIV, as well as a culture of evaluation of new policies and interventions. There is also a need for investment in capacity to analyse the increased volume of data at sub-national level so that national ‘norms’ do not lose sight of concentrations of disease, need and equity issues in particular settings.

At the same time as national capacity and capability is built, there needs to be increased support for sub national health system development in districts and cities. The front line of the organised response has been shattered from decades of vertical programs. The NCD agenda can add to this fragmentation, or alternatively take a comprehensive systems approach.

### Progressive implementation of the 25×25 agenda

As the WHO Action Plan notes [2], countries responding to the 25×25 agenda are starting from different points. Some have almost no capacity to prevent and treat NCDs, especially those emerging from conflict, while others have some capacity, often concentrated in urban centres that may function relatively well. Thus, a universal “one-size fits all” approach will not be appropriate. Countries also vary greatly in the extent to which they have moved from a fragmented model that, at best, responds to immediate demand to one that takes a broader view, employing an integrated strategy that addresses the range of risk factors, at both individual and collective level, and all steps along the trajectory from disease detection to treatment, control, and palliative care. Where resources, both in terms of money, trained workers, and systems of governance are limited, it will be necessary to start with interventions that can be delivered at scale within resource constraints. Models of health care can be developed that involve mid-level health workers, aided by simplified guidelines, and with access to essential medicines. However, while the starting points may differ, the end point should not. The 25×25 agenda sits alongside a number of other commitments and policies. These include the International Covenant on Economic, Social and Cultural Rights, which established the commitment to progressive realisation of the right to health by each state “to the maximum of its available resources” [63] and the post-2015 agenda. The most recent document (July 2014) from the Open Working Group on the Sustainable Development Goals proposes a target to “by 2030 reduce by one-third pre-mature mortality from non-communicable diseases (NCDs) through prevention and treatment [64]…”.

Thus, while countries will have to take account of available resources, there should be a clear vision of where they want to be and what can be achieved as resources increase, leading ultimately to more comprehensive, multi-faceted, multi-sectoral and development-oriented approaches integrating health and sustainability.

## Summary

In this paper we argue for the need to go beyond the ‘standard model’ of preventing NCDs, focussing only on four risk factors (plus essential medicines for people at high risk of CVD) and four diseases. We do not offer a fully-developed strategy; rather, we argue that such a strategy is needed and highlights some key features that it should contain. These include: a) balancing collective and individual responses in ways that protect health at the individual level and view health as a *common good*; b) identifying effective approaches tailored to LMICs rather than exporting questionable strategies developed in HICs; c) strengthening health systems to support prevention and treatment efforts, with an emphasis on primary health care; d) creating the ability to react in real time to emerging threats, such as the complex adaptive behaviours of the global food, tobacco, alcohol and transport industries; e) adopting measures that promote sustainable development, thereby advancing the post-2015 agenda; f) supporting prioritisation processes within health systems that respond to the local disease burden, what the country can afford, and the level of development assistance, and that are informed by but not overridden by global priorities; g) change from a universal “one-size fits all” approach to a progression from relatively simpler, public health oriented and affordable approaches to more complex, multi-sectoral and development oriented approaches. If these issues are taken into account, the chances of success may be considerably improved.
